# Supplementary material for: Prevalence of reproductive tract infections including sexually transmitted infections among married women in urban and peri-urban mid to low socioeconomic neighbourhoods of Delhi, North India: an observational study protocol
Source: BMJ Open. 2022 Mar 18;12(3):e059583. doi: 10.1136/bmjopen-2021-059583 (PMC8935169; doi:10.1136/bmjopen-2021-059583)
Supplement: Author's manuscript [file bmjopen-2021-059583.draft_revisions.pdf]

BMJ Open is committed to open peer review. As part of this commitment we make the peer review history of every article we publish publicly available.

When an article is published we post the peer reviewers' comments and the authors' responses online. We also post the versions of the paper that were used during peer review. These are the versions that the peer review comments apply to.

The versions of the paper that follow are the versions that were submitted during the peer review process. They are not the versions of record or the final published versions. They should not be cited or distributed as the published version of this manuscript.

BMJ Open is an open access journal and the full, final, typeset and author-corrected version of record of the manuscript is available on our site with no access controls, subscription charges or pay-per-view fees (http://bmjopen.bmj.com).

If you have any questions on BMJ Open's open peer review process please email info.bmjopen@bmj.com

BMJ Open

Prevalence of reproductive tract infections including sexually transmitted infections among women in urban and peri urban mid to low socioeconomic neighbourhoods of Delhi, North India an observational study

Journal:	*BMJ Open*
Manuscript ID	bmjopen-2021-059583
Article Type:	Protocol
Date Submitted by the Author:	26-Nov-2021

Complete List of Authors:	Dhabhai, Neeta; Centre for Health Research and Development Society for Applied Studies<br>Chaudhary, Ritu; Centre for Health Research and Development Society for Applied Studies, Society for Applied Studies<br>Wi, Teodora; World Health Organization<br>Mburu , Gitau ; World Health Organization, 5. Department of Sexual and Reproductive Health and Research (SHR)<br>Chowdhury, Ranadip; Centre for Health Research and Development Society for Applied Studies,<br>More, Deepak; Centre for Health Research and Development Society for Applied Studies,<br>Chatterjee, Leena; Dr Dangs Lab LLP<br>De, Devjani; Dr Dangs Lab LLP<br>Kabra, Rita ; World Health Organization, Reproductive Health and Research<br>Kiarie, James; World Health Organization<br>Habib, Ndema; World Health Organization, Department of Sexual and Reproductive Health and Research including UNDP/UNFPA/UNICEF/WHO/World Bank Special Programme of Research, Development and Research Training in Human Reproduction (HRP)<br>Dang, Arjun; Dr Dangs Lab LLP<br>Dang, Manvi; Dr Dangs Lab LLP<br>Mazumder, Sarmila; Centre for Health Research and Development Society for Applied Studies

Keywords:	REPRODUCTIVE MEDICINE, SEXUAL MEDICINE, Community gynaecology < GYNAECOLOGY

SCHOLARONE™
Manuscripts

Prevalence of reproductive tract infections including sexually transmitted infections among women in urban and peri urban mid to low socioeconomic neighbourhoods of Delhi, North India an observational study

Neeta Dhabhai[1], Ritu Chaudhary[1], Teodora Elvira WI[2], Mburu Gitau[2], Ranadip Chowdhury[1], Deepak More[1], Leena Chatterjee[3], Devjani De[3], Rita Kabra[2], James Kiarie[2], Ndema Abu Habib[2], Arjun Dang[3], Manvi Dang[3], Sarmila Mazumder[1]

[1]Centre for Health Research and Development, Society for Applied Studies, New Delhi, India.

[2] World Health Organization, Geneva, Switzerland

[3]Dr. Dangs Lab, New Delhi, India

Corresponding author

Sarmila Mazumder, Senior Scientist, Centre for Health Research and Development, Society for Applied Studies, 45 Kalu Sarai, New Delhi-110016, India. Email: sarmila.mazumder@sas.org.in

Tel: +91 11 46043751-55; Fax: +91 11 46043756

Word count: Abstract: 300; Manuscript: 3090; References: 26; Tables: 1

For peer review only

ABSTRACT

Introduction

The Global Health Sector Strategy (GHSS) on sexually transmitted infections (STI), endorsed by the World Health Assembly in 2016 aims to end STIs as public health threat by 2030. WHO conducts global estimates of prevalence to monitor progress towards achieving the same. However, limited laboratory confirmed data exists of STIs and reproductive tract infections (RTI) apart from few prevalence surveys among key populations and clinic-based reports, including in India. Syndromic approach is the cornerstone of RTI/STI management and to maximize the diagnostic accuracy, there is a need to determine the main aetiologies of vaginal discharge. The study aims to estimate the prevalence of common STIs and RTIs and their aetiological organisms in symptomatic and asymptomatic women living in the urban and peri-urban, mid- to low-socioeconomic neighbourhoods of Delhi, North India.

Methods and analysis

A cross-sectional study will be conducted among 440 married women who participated in the 'Women and Infants Integrated Interventions for Growth Study (WINGS). Information on socio-demographic profile, sexual and reproductive health will be collected, followed by examination and collection of vaginal swabs for Nucleic acid amplification tests (NAAT) to diagnose Neisseria gonorrhoeae, Chlamydia trachomatis, and Trichomonas vaginalis and for microscopy to identify Bacterial Vaginosis and Candida albicans. Treatment will be as per the syndromic approach recommendations in the National Guidelines. Data will be analysed to estimate prevalence, and factors including the presence of symptoms and signs associated with laboratory confirmed RTIs/STIs using STATA version 16.0 (StataCorp).

Ethics and dissemination

This study protocol has been approved by the ethics review committees of the World Health Organization and Society for Applied Studies (SAS/ERC/RHR-RTI/STI/2020). Approval has been obtained by the WINGS investigators from SAS ERC to share the contact details of the

For peer review only - http://bmjopen.bmj.com/site/about/guidelines.xhtml

participants with the investigators. The findings will be published in peer-reviewed journals and disseminated through scientific conferences.

Trial Registration: CTRI/2020/03/023954

For peer review only

Strengths and limitations of this study

- Limited data exists related to aetiologies of symptomatic RTI/STI in India; this study responds to the research gap.
- A key strength of this study is determining the proportion of women with symptomatic and asymptomatic RTI/STI to inform STI management and provide an indication of general population prevalence of NG, CT and TV to contribute to national and global STI estimates.
- This study responds to WHO's recommendation to determine the ethology of vaginal discharge syndrome every 2 to 3 years in-order to update or modify the national guideline.
- The whole spectrum of RTI/STIs (aerobic and viral infections) was not evaluated.
- This is not a community-based prevalence survey, which will require randomized sampling and will be costly.

For peer review only

INTRODUCTION

Sexually transmitted infections (STI) and reproductive tract infections (RTI) are a significant cause of global burden of disease.[1] Of the eight pathogens of highest public health importance, four are curable, Treponema pallidum (TP), Neisseria gonorrhoeae (NG), Chlamydia trachomatis (CT), and Trichomonas vaginalis (TV) while the other four of viral aetiology are not, Hepatitis B, Herpes simplex virus (HSV), Human Immunodeficiency Virus (HIV), and Human papillomavirus (HPV).[2 3] Globally, 374 million new infections of the four curable STIs occur annually.[2 3] These infections have significant public health consequences including infertility,[4 5] ectopic pregnancies[6 7] prematurity and neonatal deaths,[8] as well as increased risk of HIV acquisition.[9 10] Among RTIs, BV is of emerging importance as it reflects the dysbiotic state of the vaginal microbiome and increases susceptibility to other STIs.[3 4] Candidial genital infection is the leading cause of fungal vulvo-vaginitis and asymptomatic microorganism colonization can occur in up to 60% of the cases.[8]

Given the importance of STIs and RTIs, strengthening epidemiological data to inform management and control is imperative. In its global health strategy for the control of STIs and tool for strengthening surveillance of STIs, the World Health Organization (WHO) emphasizes on four key elements, including case reporting, prevalence assessment, aetiologies, and monitoring of antimicrobial resistance[11]. Global control and prevention of STIs will contribute to progress towards achievement of the Sustainable Development Goals to ensure universal access to sexual and reproductive health-care services.[2]

To address the limited national data, WHO developed the STI spectrum model to assist countries to estimate the prevalence. Recent estimates suggest that incidence of STIs has plateaued at 1 million cases annually,[9] however, significant regional heterogeneity regarding the burden and availability of data exists especially in Asia and Oceania.[9] In India, there is no recent data to determine the magnitude of STIs. Few prevalence surveys among key populations and pregnant women exist, to inform national STI estimates. Regular case reporting of infections

For peer review only

such as gonorrhoea is available, but are under-reported, thus the generation of more accurate prevalence data among the general population is needed. Apart from regular case reporting, WHO recommends studies to determine local aetiologies of STIs every 2 to 3 years to improve management.[10]

In India, it is estimated that 6% of the adult population is infected with one or more STIs at any one time. Due to limited access and affordability of STI laboratory diagnosis at the primary level of care where majority of STI cases seek care, syndromic case management remains the cornerstone of STI control.[12] This, as is the case in many low- and middle-income countries is partly due to its lower infrastructural/equipment investments and limited availability of expertise. Syndromic approach is based on presence of sign and symptoms reported and provides immediate treatment for the most common organisms causing the identified syndrome, through the use of pre-packaged colour coded drug kits. It is also recommended for the pregnant women.

Based on new evidence WHO recommends quality-assured molecular assays for treatment of people with symptoms of NG, CT and TV. If quality-assured molecular assays are not available, treatment based syndromic approach is recommended.[13] However, syndromic approach has certain limitations. It results in over treatment or missed diagnosis of cervical infections (NG and/or CT) compared with aetiological approach using molecular assay.[14 15] While aetiological determination of STI is more accurate, it is often not feasible in most health facilities. Aetiological data among symptomatic and asymptomatic women is inadequate across India.[14] Of the few studies that have explored aetiologies of vaginal discharge, the focus has been among sex workers in 2003 and 2011,[14 15] and these studies found high rates of STIs, especially NG and CT. In 2009, a comparative study of syndromic and etiologic diagnosis of STIs was conducted among general population in Delhi[16] that included symptomatic and asymptomatic pregnant and non-pregnant women. It revealed a high proportion of women complaining of vaginal discharge, self-reporting of morbidity (65%) and presence of STD-related syndrome

For peer review only

(71.4%). In the aetiological microorganism was established in 32.2% of the cases. Our study intends to build upon these findings to generate more detailed quality-assured data related to aetiologies of RTI/STIs among symptomatic and asymptomatic women.

For peer review only

METHODS

Aims and objectives

The overall aim of the study is to generate information and data related to RTI/STIs among women in India. More specifically, there are three objectives. First, to determine the prevalence of NG, CT, TV, BV and Candida albicans (CA) among women residing in the peri-urban and urban mid to low socioeconomic areas of South Delhi. Second, to assess the diagnostic validity of the current RTI/STI syndromic case management practice relative to the etiologic diagnosis based on laboratory confirmation of NG*, CT, TV, BV and CA* among symptomatic and asymptomatic women. Third, to identify demographic, behavioral, sexual and clinical factors associated with laboratory confirmed RTI/STI among women. Similar data on Syphilis is already being collected as a part of WINGS.[17]

Study design and participants

This is a cross sectional independent study enrolling women at least 14 days after their exit from WINGS. Women will be exiting WINGS at one of two time points: either at 18 months since enrolment for women who do not get pregnant, or at 24 months post-partum for women who get pregnant. The WINGS protocol was published elsewhere.[17]

Sample size

Overall, 6% of adults in India are estimated to have STIs.[14] Existing studies suggest that 32% of cases have laboratory confirmed STI.[16]Therefore, a sample size of 440 women will be adequate to ascertain the prevalence of aetiological diagnosis of STI in symptomatic and asymptomatic women with 95% confidence, 15% relative precision and 20% non-response rate allowance.

Study procedures

Recruitment

Study participants who exit forms WINGS will be contacted after at least 14 days to ascertain their willingness to participate in the and those who agree, will be offered to take part in the study.

For peer review only

Informed consent

Written individual informed consent will be obtained from all study participants at enrolment for quantitative data collection, collection of endocervical swabs and for interviews by research assistants at the study clinic. For those who are unable to read, the consent form will be read out by the worker. Those participants who are unable to sign, a thumb imprint will be taken witnessed (counter signed) by an impartial literate witness. The informed consent form will be translated into simple Hindi language that can be easily read and understood.

Screening for sampling eligibility

Women, who consent, will be screened to assess eligibility for enrolment. Participants will be asked about their last menstrual period (LMP) to check for ongoing periods, vaginal douching, insertion of any vaginal tablet or cream and sexual intercourse in previous 48 hours. A visit will be rescheduled if they confirm any of the above as blood, semen or medication can potentially interfere with test results.

Data collection

Using a structured questionnaire, the research assistants will conduct face-to-face interviews to collect information on socio-demographic, economic and sexual behaviour, symptoms of RTI/STI in previous one week, history of RTI/STIs and treatment received during last three months, among other data (questionnaire provided in Supplemental file).

Gynaecological examination

A thorough history and genital and pelvic examination including abdominal palpation will be done by the study gynaecologist with aseptic precautions in complete privacy, for all participants irrespective of the symptoms. With the participant in the dorsal lithotomy position, a visual inspection of the external genitalia, followed by a per speculum and bimanual examination will be done.

Signs of RTI/ STI and other observations will be documented in an electronic tracker. These will include nature of vaginal discharge - colour, odour, consistency, amount, presence and type of

For peer review only

cervical discharge, condition of cervix, any genital ulcers, adnexal or pelvic tenderness, size of uterus, any adnexal mass or lump. High vaginal swab and endo-cervical swab will be collected as per the standard operating procedures. Women will be provided free medication according to the diagnosis, as per the National Guidelines on RTI/STI.[18]

Sample collection, storage and transportation

Two swabs will be collected, one vaginal and other endocervical, by the study gynaecologist. The vaginal walls and cervix will be visualized using self-retaining sterile Cusco's speculum. Vaginal swab specimen will be collected from the posterior fornix and the lateral walls of the vagina for detection of BV and CA and kept in a pre-labelled dry collection tube. Following this, the endocervical swab from the test kit will be inserted gently into the endocervical canal, rotated and withdrawn and kept in the pre-labelled transport reagent container for Nucleic Acid Amplification test (NAAT) for CT, NG and TV.

All samples will be stored at 2-8°C in the laboratory and transportation to the testing laboratory will be done daily, in cool boxes maintained at 2-8°C.

Laboratory procedures

NAAT, a real time polymerase chain reaction (PCR) having the highest specificity and sensitivity (98-100%) among the available conventional tests, will be used to detect CT, NG and TV.[19] We will use Cartridge Based (CB) NAAT Gene Xpert platform which is an automated in vitro diagnostic test for qualitative detection and differentiation of DNA of CT, NG and TV and is performed on Cepheid GeneXpert Instrument Systems.[20] With this we will use the GeneXpert CT/NG Cepheid In vitro diagnostic (IVD), and GeneXpert TV Cepheid IVD kits.[21]

The GeneXpert Instrument Systems automate and integrate DNA extraction, amplification, and detection of the target sequences in simple or complex samples using Reverse transcription (RT-PCR). The systems consist of an instrument connected with a computer, with preloaded software for running tests and viewing and generating results. The systems require single-use disposable cartridges that hold the PCR reagents and host the PCR process.

For peer review only

Reagents for the detection of a Sample Processing Control (SPC), a Sample Adequacy Control (SAC), and a Probe Check Control (PCC) are also included in the cartridge. The SPC is present to control for adequate processing of the target bacteria and to monitor the presence of inhibitors in the PCR reaction.

The SAC reagents detect the presence of a single copy human gene and monitor whether the sample contains human DNA. The PCC verifies reagent rehydration, PCR tube filling in the cartridge probe integrity, and dye stability. The primers and probes in the Xpert CT/NG test detect chromosomal sequences in the bacteria. One target is detected for CT (CT1) and two different targets are detected for NG (NG2 and NG4). Both NG targets need to be positive for the Xpert CT/NG test to return a positive NG result. Prepared sample is transferred to the sample chamber of the Gene expert cartridge and loaded on the GeneXpert machine and the test is performed.

Summary and detailed test results are generated by the software in approximately 90 minutes and are displayed in tabular and graphic formats. Nugent's Scoring criteria using a gram stained slide is the current 'gold standard with ~77% specificity and 90% sensitivity will be used to diagnose BV.[22]

A smear will be prepared and gram staining done. Microscopic examination will be done to detect clue cells and semi-quantitative evaluation for Gram-positive, Gram-negative or Gram-variable bacterial morphotypes quantified. Women will be categorized as non-BV (Nugent score 0–3), intermediate (Nugent score 4–6) or BV (Nugent score ≥ 7) (Table 1).

Table 1. Nugent Score

Organism morphotype	Number/oil immersion field	Score
Lactobacillus-like (parallel sided, gram positive rods)	>30	0
	5-30	1
	1-4	2
	<1	3
	0	4

**Organism morphotype**	**Number/oil immersion field**	**Score**
Mobiluncus-like (curved, gram negative rods)	>5	2
	<1.4	1
	0	0
Gardnerella/Bacteroides-like (tiny, gram variable coccobacilli and pleomorphic rods with vacuoles	>30	4
	5-30	3
	1-4	2
	<1	1
	0	0

Total score: 0-3=Normal; 4-6=Intermediate, repeat test later; 7-10= Bacterial vaginosis

Microscopical examination of the vaginal smear will be done for yeast cells with pseudo hyphae to detect CA.

Quality Control

For Gram stain, on each slide, a control area for Staph aureus (ATCC25923) & E. coli (ATCC25922) is put as a staining control. For CBNAAT, each test kit includes an inbuilt Sample Processing Control (SPC), a Sample Adequacy Control (SAC) and a Probe Check Control (PCC). External controls (one positive and one negative) will be used in accordance with local, state accrediting organizations, as applicable.

Kit Verification

As the Cepheid kits to be used for the study for CT/NG are FDA approved while that for TV is Research Use Only (RUO), a kit verification procedure is planned to evaluate the performance of the GeneXpert platform. A verification panel comprising of 17 samples containing known CT, NG, and TV of high, medium, and low concentration will be tested on GeneXpert using the recommended kits. The results will be compiled and reviewed to determine the suitability of the platform and the kits for diagnosis of STI in the study.

For peer review only

Management of STIs

Women with signs and symptoms of STI will be treated based on the syndromic approach as recommended in the national guidelines.[11]Their partners will be notified and provided necessary treatment. The laboratory reports will be reviewed by the study gynaecologists and delivered to their homes with advise for revisit if needed. Asymptomatic women who need further treatment will be invited to the study clinic for an appointment with the study gynaecologists and to receive medicines. Women will also be provided information and counselled on prevention of STI/RTI. Women requiring admission or additional management will be referred to the collaborating hospital. A complete course of treatment and supply of condoms will be provided for free.

Statistical analysis and outcome measures

Descriptive and inferential statistical analysis will be performed. Simple proportions and 95% confidence bounds will be calculated to determine prevalence for overall laboratory confirmed RTI/STI and individual micro-organism by binomial exact method. We will use a method of purposeful selection of covariates to identify variables for the multivariable models.[23 24] In the multivariable models, we will include the variables that will change the odds ratio of the outcome variables by 20% from the univariable models. We will present the adjusted models, including variables that will be identified in the procedure. We will assess whether any of the demographic, behavioural, sexual and clinical risk factors are modifying the association between laboratory conformed RTI/STI and any of the above-mentioned variables on a multiplicative Computation of sensitivity, specificity, predictive values and likelihood ratios will be derived in diagnosing vaginal infection (TV, BV) and cervical infection (NG, CT) using NAAT as the gold standard of diagnosis compared with syndromic approach which is informed by the presence of symptoms and signs. Analysis will be done using STATA, version 16.0 (StataCorp).

For peer review only

Patient and public involvement

Participants and the public were not involved in the design of this study. However, study results will be shared with the participants and informed about their aetiological diagnosis and provided appropriate treatment.

Confidentiality

All data will be de-identified, analyzed and aggregated before reporting. Furthermore, data will be stored securely and only reviewed by the immediate research team. The information collected from the study participants will be kept confidential and stored in a locked area which can only be accessed by the study team. The participants will not be identified by his/her name but only by a study number. All study documents will be stored for a period of 5 years after which they will be destroyed. Knowledge from this study will be disseminated through reports given to the ethics review committee, government agencies and publications but none will have the name of the study participants.

Tokens/compensation

Participants will be provided with a reasonable token (not exceeding USD 2) of appreciation, along with free medical examination,treatment for STI/RTI and condoms. Usually this is given to compensate for the time spent on study participation. This is considered reasonable to compensate participants for their time. The amount is not considered large to constitute an undue inducement.

Gender and social equity considerations

The proposed study addresses the sexual and reproductive rights of women related to RTI/STI and will identify risks and vulnerabilities in relation to RTI/ STIs in the study context. By understanding the prevalence of laboratory confirmed RTI/STIs as well as diagnostic validity of syndromic approach, the study will provide useful information to the national program. It will also assist women to access treatment, including asymptomatic women, which will be useful in addressing inequality to service access. The research questions are focused on women.

For peer review only

However, there will be opportunities for sexual partners of women identified with RTI/STI to be notified and subsequently provided with treatment as per syndromic management following laboratory/ etiologic results.

Community engagement

This study will enrol women who previously participated in WINGS, which has a large component of community participation. Participants will also be contacted at their homes, and local communities will continually be reached and mobilized with the results to enhance uptake of interventions that promote better woman and infant outcomes.

Research capacity strengthening

It is envisioned that exchange of information related to conduct of research and data analysis will continually take place between WHO and the research partners.

Dissemination

The result of the study will be presented in scientific conferences and published in appropriate peer-reviewed journals.

For peer review only

DISCUSSION

This study aims to fill an existing gap related to lack of epidemiologic information on STIs in India. A recent systematic review of syndromic approach found that there were very limited validation of syndromic approach as well as etiologic studies globally.[25] With an ever-changing epidemiology of STIs, there is a need to encourage countries to regularly conduct etiologic studies of syndromes, to inform national STI case management guidelines.

This study will evaluate the diagnostic validity of RTI/STI syndromic approach among women of reproductive age in India while providing important information related to the prevalence of RTI/STIs and the aetiologies of vaginal discharge syndrome in the study context, where there is insufficient prevalence and etiologic data. Given the serious consequences on women's health, sexual and social relationships and psychological well-being[26] this research will make an important contribution to the understanding and mitigation of RTI/STIs in India. Data on prevalence will be relevant for improving the national STI case management in India, and for planning RTI/STI services for women to reduce maternal and neonatal morbidity and mortality, adverse birth outcomes, and the sequelae of these infections.

In context of high volume of specimens, one of the key challenges for this study will be to ensure quality of sample collection, transport and processing for which through training and regular monitoring will be done. While important, our primary intention at this point is not to compare RTI/STI prevalence between two groups of women (those who get pregnant and those who do not). This sub-study will not collect data related to syphilis since prevalence data for on Syphilis is already being collected as a part of WINGS.[16]

For peer review only

Abbreviations

GHSS; Global Health Sector Strategy, **RTI;** Reproductive tract infections, **STI;** Sexually transmitted infections, **WINGS;** Women and Infants Integrated Interventions for Growth Study, **NABL;** National Accreditation Board for Testing and Calibration, **NAAT;** Nucleic acid amplification tests, **NG;** Neisseria gonorrhoeae, **CT**; Chlamydia trachomatis, **TV**; Trichomonas vaginalis, **BV;** Bacterial Vaginosis, **CA;** Candida albicans, **TP;** Treponema pallidum, **HSV**; Herpes simplex virus, **HIV;** Human Immunodeficiency Virus, **HPV;** Human papillomavirus, **CG;** Candidial genital, **WHO;** World Health Organization, **LMP;** last menstrual period, **NAAT;** Nucleic Acid Amplification test, **PCR;** Polymerase chain reaction, **SPC;** Sample Processing Control, **SAC;** Sample Adequacy Control, **PCC;** Probe Check Control, **RUO;** Research use only, **ERC:** Ethics Research Committee.

Acknowledgements

This study is funded by WHO Department of Sexual and Reproductive Health and Research. WHO/SRH department includes the UNDP-UNFPA-UNICEF-WHO-World Bank Special Programme of Research, Development and Research Training in Human Reproduction (HRP). Authors thank Dr Dang's laboratory, implementation partners of the WINGS study (Bills and Melinda Gates Foundation (BMGF), the department of Biotechnology and the Government of India through Biotechnology Industry Research Assistance Council (BIRAC) including CHRD-SAS, Safdarjung Hospital and the WHO Department of Maternal, Newborn, Child and Adolescent health (MCA). Views expressed are those of the authors and not necessarily those of their respective institutions.

Authors' contributions

JK, TAW, GM, RK, SM, ND, conceived the study. TW, GM, SM, ND, RC, NDH, NAH and DM prepared the protocol. All authors reviewed and approved the final manuscript.

For peer review only

Funding

This study is funded by WHO Department of Sexual and Reproductive Health and Research. WHO/SRH Department includes the UNDP-UNFPA-UNICEF-WHO-World Bank Special Programme of Research, Development and Research Training in Human Reproduction (HRP). The funding agency has no role in the design of the current protocol and will not have any role in the implementation, analysis and reporting of the study results.

Competing interests

None declared

Patient consent for publication

Not required

Ethics and dissemination

Approval for this study has been obtained from the Ethics Review Committee of the Centre for Health Research and Development Society for Applied Studies (SAS/ERC/RHR-RTI/STI/2020) and the World Health Organization, Geneva (A-ID: A65999). Approval has been obtained by the WINGS investigators from SAS ERC to share the contact details of the participants with the investigators. The findings will be published in peer-reviewed journals and disseminated through scientific conferences.

Data availability statement

The data underlying this article will be shared on reasonable request to the corresponding author. Data arising from the study will be kept by SAS for 5 years and anonymized forms of these data will be available on request from the corresponding author.

Declaration of interests

The authors declare no competing interests.

For peer review only

REFERENCES

1. WHO. Report on global sexually transmitted infection surveillance, 2018. Geneva: World Health. 2018

2. World Health Organization. Global progress report on HIV, viral hepatitis and sexually transmitted infections, . *Accountability for the global health sector strategies 2016–2021* 2021

3. Newman L, Rowley J, Vander Hoorn S, et al. Global Estimates of the Prevalence and Incidence of Four Curable Sexually Transmitted Infections in 2012 Based on Systematic Review and Global Reporting. *PLOS ONE* 2015;10(12):e0143304. doi: 10.1371/journal.pone.0143304

4. Bautista CT, Wurapa E, Sateren WB, et al. Bacterial vaginosis: a synthesis of the literature on etiology, prevalence, risk factors, and relationship with chlamydia and gonorrhea infections. *Mil Med Res* 2016;3:4-4. doi: 10.1186/s40779-016-0074-5

5. Bertini M, Serdaroglu S, Kutlubay Z. Bacterial vaginosis and sexually transmitted diseases: relationship and management. *Fundamentals of Sexually Transmitted Infections* 2017;75

6. Etiological study of vaginal discharge syndrome in RTI clinic attendees in a tertiary care hospital; 2017.

7. Siracusano S, Silvestri T, Casotto D. Sexually transmitted diseases: epidemiological and clinical aspects in adults. *Urologia* 2014;81(4):200-8. doi: 10.5301/uro.5000101 [published Online First: 2014/12/24]

8. Sustr V, Foessleitner P, Kiss H, et al. Vulvovaginal Candidosis: Current Concepts, Challenges and Perspectives. *Journal of fungi (Basel, Switzerland)* 2020;6(4) doi: 10.3390/jof6040267 [published Online First: 2020/11/12]

For peer review only

9. Rowley J, Vander Hoorn S, Korenromp E, et al. Chlamydia, gonorrhoea, trichomoniasis and syphilis: global prevalence and incidence estimates, 2016. *Bulletin of the World Health Organization* 2019;97(8):548-62p. doi: 10.2471/blt.18.228486 [published Online First: 2019/08/07]

10. Organization WH. Strategies and laboratory methods for strengthening surveillance of sexually transmitted infections. *Switzerland, Geneva: WHO Website* 2012

11. World Health Organization. A tool for strengthening STI surveillance at the country level. 2015

12. National guidelines on prevention, management and control of reproductive tract infections including sexually transmitted infections.2006 http://naco.gov.in/sites/default/files/National_Guidelines_on_PMC_of_RTI_Including_STI%201.pdf

13. World Health Organization. Guidelines for the management of symptomatic STIs. Geneva, WHO. 2021

14. Desai VK, Kosambiya JK, Thakor HG, et al. Prevalence of sexually transmitted infections and performance of STI syndromes against aetiological diagnosis, in female sex workers of red light area in Surat, India. *Sexually transmitted infections* 2003;79(2):111-5. doi: 10.1136/sti.79.2.111 [published Online First: 2003/04/12]

15. Das A, Prabhakar P, Narayanan P, et al. Prevalence and assessment of clinical management of sexually transmitted infections among female sex workers in two cities of India. *Infectious diseases in obstetrics and gynecology* 2011;2011:494769. doi: 10.1155/2011/494769 [published Online First: 2011/07/13]

16. Ray K, Muralidhar S, Bala M, et al. Comparative study of syndromic and etiological diagnosis of reproductive tract infections/sexually transmitted infections in women in Delhi. International journal of infectious diseases : IJID : official publication of the International

For peer review only

Society for Infectious Diseases 2009;13(6):e352-9. doi: 10.1016/j.ijid.2008.11.021 [published Online First: 2009/02/25]

17. Taneja S, Chowdhury R, Dhabhai N, et al. Impact of an integrated nutrition, health, water sanitation and hygiene, psychosocial care and support intervention package delivered during the pre- and peri-conception period and/or during pregnancy and early childhood on linear growth of infants in the first two years of life, birth outcomes and nutritional status of mothers: study protocol of a factorial, individually randomized controlled trial in India. *Trials* 2020;21(1):127. doi: 10.1186/s13063-020-4059-z [published Online First: 2020/02/02]
18. Welfare, D.o.A.C.M.o.H.a.F. and G.o. India, National Guidelines on Prevention, Management and Control of Reproductive Tract Infections and Sexually Transmitted Infections. . 2014 doi: http://naco.gov.in/sites/default/files/National%20RTI%20STI%20technical%20guidelines%20Sep2014_0.pdf
19. WHO laboratory validation of Xpert® CT/NG and Xpert® TV on the GeneXpert system verifies high performances 2018 doi: https://doi.org/10.1111/apm.12902
20. CBNAAT Gene Xpert platform is an automated in vitro C and differentiation of DNA from CT, NG. https://www.cepheid.com/en/systems/GeneXpert-Family-of-Systems/GeneXpert-System
21. The GeneXpert CT/NG Cepheid IVD, and GeneXpert used to detect CT, NG, and TV. https://www.cepheid.com/en/tests/Sexual-Health/Xpert-CT-NG
22. Cox C, McKenna JP, Watt AP, et al. New assay for Gardnerella vaginalis loads correlates with Nugent scores and has potential in the diagnosis of bacterial vaginosis. Journal of medical microbiology 2015;64(9):978-84. doi: 10.1099/jmm.0.000118 [published Online First: 2015/08/25]

For peer review only

23. Bursac Z, Gauss CH, Williams DK, et al. Purposeful selection of variables in logistic regression. *Source code for biology and medicine* 2008;3:17. doi: 10.1186/1751-0473-3-17 [published Online First: 2008/12/18]

24. Hosmer Jr DW, Lemeshow S, Sturdivant RX. Applied logistic regression: John Wiley & Sons 2013.

25. Zemouri C, Wi TE, Kiarie J, et al. The Performance of the Vaginal Discharge Syndromic Management in Treating Vaginal and Cervical Infection: A Systematic Review and Meta-Analysis. *PLoS One* 2016;11(10):e0163365. doi: 10.1371/journal.pone.0163365 [published Online First: 2016/10/06]

26. Cooperman NA, Shastri JS, Shastri A, et al. HIV prevalence, risk behavior, knowledge, and beliefs among women seeking care at a sexually transmitted infection clinic in Mumbai, India. *Health care for women international* 2014;35(10):1133-47. doi: 10.1080/07399332.2013.770004 [published Online First: 2013/05/11]

For peer review only

	LETTER OF APPROVAL BY ERC FOR STUDIES NOT REGULATED BY CDSCO	SOP Number	SAS/SOP/ERC/008T4
		Version Number	1.0
		Effective Date	30/NOV/2019
		Revision Due Date	29/NOV/2021

Ethics Review Committee
Centre for Health Research and Development
Society for Applied Studies
45, Kalu Sarai, New Delhi-110016, India

For peer review only

To,
Dr. Sarmila Mazumder
Principal Investigator
Centre for Health Research and Development
Society for Applied Studies
45, Kalu Sarai,
New Delhi-110016, India

Dr. Neeta Dhabhai
Principal Investigator
Centre for Health Research and Development
Society for Applied Studies
45, Kalu Sarai,
New Delhi-110016, India

Ref No: SAS/ERC/RHR-RTI/STI/2020 Date: 10/JAN/2020

Dear Dr. Mazumder and Dr. Dhabhai,

The Ethics Review Committee had reviewed your application to conduct the research study **titled "An ancillary study to the WINGS on reproductive tract infections/ sexually transmitted infections among urban and peri urban women in North India"** in a full committee review meeting conducted on 02/JAN/2020.

The following documents were reviewed:

1. RTI/STI study proposal version 1.0 dated 28/Nov/2019
2. Full review form dated 28/Nov/2019
3. Undertaking by the Investigator dated 28/Nov/209
4. Participant information sheet and informed consent forms version 1.0 dated 28/Nov/2019 (English)
5. Participant information sheet and informed consent forms version 1.0 dated 28/Nov/2019 (Hindi)
6. Translation and back translation certificates
7. CVs of the investigators

Level of risk	Less than minimal risk	☑	Minimal	☐	More than minimal risk	☐
Type of review	Exempt from review	☐	Expedited	☐	Full committee	☑

For peer review only - http://bmjopen.bmj.com/site/about/guidelines.xhtml

	LETTER OF APPROVAL BY ERC FOR STUDIES NOT REGULATED BY CDSCO	SOP Number	SAS/SOP/ERC/008T4
		Version Number	1.0
		Effective Date	30/NOV/2019
		Revision Due Date	29/NOV/2021

The following members of the Ethics Review Committee were present at the meeting held on 02/JAN/2020, 3:45pm at 45, Kalu Sarai, New Delhi – 110 016.

1. Dr. Binoo Nayar, Chairperson, Member, Clinician
2. Ms. Swati Naik, Acting Member Secretary, Member, Community Representative
3. Dr. Jeeva Sankar, Member, Basic Medical Scientist
4. Colonel Gian Sagar, Member, Legal Expert
5. Dr. Sanghmitra Acharya, Member, Social Scientist
6. Dr. Satinder Aneja, Member, Clinician (Observer)

We approve the trial to be conducted in its presented form.

Initial approval date: 10/JAN/2020
Approval expiration date: 09/JAN/2021

The total number approved for enrollment is 440 women.

Following are the Principal Investigator's Responsibilities with respect to continuous reporting to ERC:

- The Ethics Review Committee expects to be informed about the progress of the study. A status report must be submitted (at least 2 weeks in advance of the approval expiration date) reviewed and approved by the ERC before the current approval expires, or at least annually from the date of this approval, whichever is earlier.
- The PI shall submit / inform (as applicable) any relevant changes that may affect the study in its presented form to ERC for review / notification as applicable, along with relevant documents. Changes or modifications to this research activity must be submitted to and approved by the ERC before they are implemented. Unapproved changes will be considered as protocol deviations and will require reporting to Ethics Committee.
- The PI shall provide a copy of the final study report to the ERC.
- The ERC must be informed of any untoward or serious and unexpected events happening during the course of the study.

It is the responsibility of the investigator and all research staff to comply with the applicable ethical and/or regulatory guidelines governing human subject research.

For peer review only - http://bmjopen.bmj.com/site/about/guidelines.xhtml

CHRD	LETTER OF APPROVAL BY ERC FOR STUDIES NOT REGULATED BY CDSCO	**SOP Number**	SAS/SOP/ERC/008T4
		**Version Number**	1.0
		**Effective Date**	30/NOV/2019
		**Revision Due Date**	29/NOV/2021

The ERC is constituted and conforming with the requirements of ICMR's Ethical Guidelines for Biomedical Research on Human Participants, ICH Harmonised Tripartite Guideline, Guideline for Good Clinical Practice, Declaration of Helsinki. The OHRP Federal Wide Assurance number for ERC is FWA00001757, IRB 00007526.

Yours sincerely,

BINOO NAYAR
Name of Chairperson

Signature of Chairperson

10 - 01 - 2020
Date

10/01/2020
Signature of Member Secretary

For peer review only - http://bmjopen.bmj.com/site/about/guidelines.xhtml

World Health Organization

Research Ethics Review Committee (WHO ERC)

20, AVENUE APPIA – CH-1211 GENEVA 27 – SWITZERLAND – HTTP://WWW.WHO.INT/ETHICS/REVIEW-COMMITTEE/EN/ – HTTPS://EXTRANET.WHO.INT/ERCWEB/LOGIN.PHP

WHO ERC
Review Summary

Protocol ID: A65999
Country: India
Protocol Title: An ancillary study to the WINGS Study on reproductive tract infections / sexually transmitted infections among urban and peri urban women in North India
Version: 1 **Dated:** 06/11/2019
WHO Responsible Staff Member: Daniel Gitau MBURU
Responsible Unit: WHO/HQ/FWC/RHR
Meeting Date: 16/01/2020

Dear Dr. Daniel Gitau MBURU,
Please find the review summary of the Protocol "An ancillary study to the WINGS Study on reproductive tract infections / sexually transmitted infections among urban and peri urban women in North India", which was submitted to the Secretariat on 22/11/2019. This proposal underwent regular review.

The outcome of the review is provided below. When responding, please submit the following:

1. A cover memorandum that addresses your responses, POINT BY POINT, to each of the queries in sections A and B.
   Section C contains Suggestions to improve the proposal but there is no obligation to follow them.

2. An Amended protocol including the responses in bold, highlighted or in track changes. Please ensure that tracking formatting changes is switched off or that all formatting changes have been accepted and that no comments which the team may exchange during the editing are included in the track changes version. The protocol should include all relevant documentation (ICF, study instruments, peer review, etc.) even if already submitted.

Please note that comments in the introductory paragraph are meant for the WHO Responsible Staff Member, though you may decide to share them with the PI.

PLEASE RESPOND TO THIS REVIEW SUMMARY WITHIN A three MONTH PERIOD, OR PROVIDE THE ERC SECRETARIAT A VALID JUSTIFICATION FOR THE DELAY.

According to the ERC this study cannot be considered an ancillary study or a sub-study as it has no meaningful connection with the original study. The only commonality is that because of convenience, the study population from the original study is intended to be used for this study. This raises several ethical concerns. Please find specific comments below.

A. Amendments (Response and change required)

This section includes queries and comments on your protocol, study instruments or the informed consent form for which the ERC requires your response and where relevant, appropriate amendments to the protocol, study instruments or the informed consent.

1. Protocol
   - 1.1. Please provide an amended proposal specifying the version number and/or date on each page.
   - 1.2. The main ethical question with regards to this study is whether it is appropriate to ask women from the previous study to take part in a new study -at the time of their last day of participation when they think they are finishing their contribution to research. This would not only increase the burden on participation but may affect autonomy -given the relationship they have established with the research team, which may put them in a difficult position to decline participation.
     - 1.2.1. Please explain the reasons for not consenting prospective participants to the WINGS study in the new one. Given that WINGS is still undergoing, women could be invited at the time of recruitment to be part of one or both studies. Please address.

For peer review only - http://bmjopen.bmj.com/site/about/guidelines.xhtml

World Health Organization

Research Ethics Review Committee (WHO ERC)

20, AVENUE APPIA – CH-1211 GENEVA 27 – SWITZERLAND – HTTP://WWW.WHO.INT/ETHICS/REVIEW-COMMITTEE/EN/ – HTTPS://EXTRANET.WHO.INT/ERCWEB/LOGIN.PHP

1.2.2. It is understood that from the researchers perspective, the WINGS study offers the possibility of accessing a representative sample to answer their study question. However, a clear separation should be made to participants between the two studies. Please explain how this will be achieved so that it is clearly understood by participants to the WINGS study that there is no connection between the two studies, and that participants feel free to decline participation. For example, letters could be sent by the WINGS study to participants informing them of the new study, explaining that there is no relationship between the two and asking them whether they would be interested in this new study, another option is to post information on the walls of health facilities explaining the study, etc.

1.3. Please confirm that the study will take responsibility for ensuring effective treatment of participants who were detected as positive rather than simply referring them to routine services. Since the study team identified the STI, the study team is responsible for ensuring that treatment provided is effective. Please address.

2. Informed Consent Forms

2.1. Please modify based on the comments stated in Sections A.1 and B.1.

B. Clarifications (Response required but change may not be required)

This section includes queries on your protocol, study instruments or the informed consent form for which the ERC to make changes to your protocol.

1. Protocol

1.1. It is stated that the protocol is based on the WHO guidelines 2005 (Sexually transmitted and other reproductive tract infections: A guide to essential practice.) and that treatment for participants testing positive will be based on the National Guidelines 2014. However, treatment guidelines were published by WHO in 2016. Please clarify the reasons for not using the latest WHO guidelines.

1.2. Please clarify whether psycho-social support will be available in the context of counselling or by other means for women testing positive that are in need.

C. Suggestions

This section consists of suggestions for alternative scientific or technical approaches or methods for conducting the research but which do not raise critical, ethical issues. These are meant to be helpful to investigators and are presented as suggestions for you to consider incorporating into a revised protocol. No response from you is required for any comment in this section. If, however, you do make changes to the protocol as a result of these suggestions, please submit the revised protocol to the ERC.

NIL

For peer review only - http://bmjopen.bmj.com/site/about/guidelines.xhtml

World Health Organization

Research Ethics Review Committee (WHO ERC)

20, AVENUE APPIA – CH-1211 GENEVA 27 – SWITZERLAND – HTTP://WWW.WHO.INT/ETHICS/REVIEW-COMMITTEE/EN/ – HTTPS://EXTRANET.WHO.INT/ERCWEB/LOGIN.PHP

Protocol ID: A65999 **Meeting Date**: 16/01/2020

Based on the above comments, the Committee has the following recommendation(s) for this proposal:

[ ] The proposal is ***Approved as submitted.*** No modifications are required.

[X] The proposal is ***Conditionally Approved; requires amendments and/or clarifications.*** Final approval is contingent upon an adequate response by the Principal Investigator, to the satisfaction of the reviewers or the Chair on behalf of the ERC.

[ ] The proposal is ***Not approved; requires additional information and/or rewriting.*** A revised version of the proposal should be re-submitted by the WHO responsible staff member as a new submission to the ERC for re-review by Committee.

[ ] The proposal is ***Rejected***. The proposal is ethically unacceptable, for the reasons stated above. The Principal Investigator may submit a new proposal that takes into consideration the ethical issues raised by the Committee. If you do not agree with the Committee's assessment, please feel free to submit an appeal to the Chair of the ERC, through the Secretariat.

NOTE: Final Approval of the Proposal is contingent upon submission of the following:

[ ] Local ethics approval(s) [ ] Other relevant documents

The ERC would like to receive a copy of the recommendations of the local ethics committee when available.

IMPORTANT

1. *Any changes to the proposal* <u>*or*</u> *to the attachments (informed consent/study instruments etc.) should be approved by ERC before being implemented.*
2. *The approval for this proposal is valid for a period of* ***one year only.***
3. *Please resubmit this proposal for a Continuing Review at least 2 months before the next re-approval period.*

Chairperson …………………………………… **Date**. 27.01.2020

Name: Peter Olumese

**<u>FINAL APPROVAL</u>**	**<u>FOR THE SECRETARIAT</u>**
Amendments and Clarifications to the proposal have been reviewed. The protocol (Version: 4 Date: 11.04.2020) and informed consent Forms (Dated: 11.04.20 V4) submitted on 20.04.2020 are approved by the ERC<br><br>**Chairperson** ……………………………………<br>PO/AL/LO<br>**Name** ……………………………………<br>**Date** 24.04.2020……………………………	Amendments and Clarifications to be reviewed:<br>[ ] Electronically by ERC<br>[ ] by Primary reviewers<br>[ ] by Secretariat<br><br>Amendments approved / Clarifications accepted on<br>Local ERC approval(s) obtained on<br><br>18.04.2020<br><br>Relevant Documents submitted on: NA<br><br>Comments:<br>Signature Date

For peer review only - http://bmjopen.bmj.com/site/about/guidelines.xhtml

World Health Organization

COVERING LETTER
LETTRE D'ACCOMPAGNEMENT

Global Procurement and Logistics
Global Service Centre
Block 3510
Jalan Teknokrat 6
63000 Cyberjaya
MALAYSIA
gsc-procurement@who.int

WHO Reference/ *Référence OMS*

WHO Reference	2020/1026672-0
Purchase Order	202553792
Unit Reference	FWC/RHR/HRX-PS

sarmila.mazumder@sas.org.in
SOCIETY FOR APPLIED STUDIES
NEWDELHI02
Centre For Health Research and Development
45, Kalu Sarai
New Delhi
110016
India

TECHNICAL SERVICES AGREEMENT (TSA)

Re: To successfully execute the protocol for the study on reproductive tract infections / sexually transmitted infections among urban and peri urban women in North India A-ID: A65999

We are enclosing the Technical Services Agreement between the World Health Organization and SOCIETY FOR APPLIED STUDIES, NEWDELHI02 for conducting the above-mentioned work. We also enclosed three attachmen referenced in the Agreement.

Kindly acknowledge your acceptance of this contract by returning the email with a copy of duly signed Purchase Order (all pages).

For any technical or scientific questions related to this Agreement, please contact the responsible technical officer, Daniel Gitau MBURU, mburug@who.int.

On behalf of the World Health Organization, we thank you for your collaboration.

WHO Global Service Centre

Cc: WHO Representative, India

Concerne: To successfully execute the protocol for the study on reproductive tract infections / sexually transmitted infections among urban and peri urban women in North India A-ID: A65999

Veuillez trouver ci-joint l'Accord de Services Techniques entre l'Organisation Mondiale de la Santé et SOCIETY FOR APPLIED STUDIES, NEWDELHI02 vous permettant de mener à bien le travail susmentionné. Veuillez également trouver three pièces jointes mentionnées dans l'Accord.

Merci de confirmer votre acceptation de ce contrat en nous retournant le courriel et une copie dûment signee du Bon de Commande (complet)

Pour toutes questions à caractère scientifique ou technique ayant trait à cet Accord, veuillez contacter le responsible technique Daniel Gitau MBURU, mburug@who.int.

Au nom de l'Organisation Mondiale de la Santé, nous vous remercions de votre collaboration.

Centre de Soutien Administratif Mondial de l'OMS

Cc: Représentant de l'OMS, India

For peer review only

SPIRIT
STANDARD PROTOCOL ITEMS: RECOMMENDATIONS FOR INTERVENTIONAL TRIALS

SPIRIT 2013 Checklist: Recommended items to address in a clinical trial protocol and related documents*

Section/item	Item No	Description	Addressed on page number
**Administrative information**			
Title	1	Descriptive title identifying the study design, population, interventions, and, if applicable, trial acronym	1
Trial registration	2a	Trial identifier and registry name. If not yet registered, name of intended registry	3
	2b	All items from the World Health Organization Trial Registration Data Set	-
Protocol version	3	Date and version identifier	-
Funding	4	Sources and types of financial, material, and other support	17
Roles and responsibilities	5a	Names, affiliations, and roles of protocol contributors	17
	5b	Name and contact information for the trial sponsor	18
	5c	Role of study sponsor and funders, if any, in study design; collection, management, analysis, and interpretation of data; writing of the report; and the decision to submit the report for publication, including whether they will have ultimate authority over any of these activities	18
	5d	Composition, roles, and responsibilities of the coordinating centre, steering committee, endpoint adjudication committee, data management team, and other individuals or groups overseeing the trial, if applicable (see Item 21a for data monitoring committee)	-

For peer review only

**Introduction**			
Background and rationale	6a	Description of research question and justification for undertaking the trial, including summary of relevant studies (published and unpublished) examining benefits and harms for each intervention	5-7
	6b	Explanation for choice of comparators	-
Objectives	7	Specific objectives or hypotheses	8
Trial design	8	Description of trial design including type of trial (eg, parallel group, crossover, factorial, single group), allocation ratio, and framework (eg, superiority, equivalence, noninferiority, exploratory)	8
**Methods: Participants, interventions, and outcomes**			
Study setting	9	Description of study settings (eg, community clinic, academic hospital) and list of countries where data will be collected. Reference to where list of study sites can be obtained	8
Eligibility criteria	10	Inclusion and exclusion criteria for participants. If applicable, eligibility criteria for study centres and individuals who will perform the interventions (eg, surgeons, psychotherapists)	9
Interventions	11a	Interventions for each group with sufficient detail to allow replication, including how and when they will be administered	9-13
	11b	Criteria for discontinuing or modifying allocated interventions for a given trial participant (eg, drug dose change in response to harms, participant request, or improving/worsening disease)	-
	11c	Strategies to improve adherence to intervention protocols, and any procedures for monitoring adherence (eg, drug tablet return, laboratory tests)	-
	11d	Relevant concomitant care and interventions that are permitted or prohibited during the trial	13
Outcomes	12	Primary, secondary, and other outcomes, including the specific measurement variable (eg, systolic blood pressure), analysis metric (eg, change from baseline, final value, time to event), method of aggregation (eg, median, proportion), and time point for each outcome. Explanation of the clinical relevance of chosen efficacy and harm outcomes is strongly recommended	-
Participant timeline	13	Time schedule of enrolment, interventions (including any run-ins and washouts), assessments, and visits for participants. A schematic diagram is highly recommended (see Figure)	8

For peer review only - http://bmjopen.bmj.com/site/about/guidelines.xhtml

Sample size	14	Estimated number of participants needed to achieve study objectives and how it was determined, including clinical and statistical assumptions supporting any sample size calculations	8
Recruitment	15	Strategies for achieving adequate participant enrolment to reach target sample size	8
**Methods: Assignment of interventions (for controlled trials)**			
Allocation:			
Sequence generation	16a	Method of generating the allocation sequence (eg, computer-generated random numbers), and list of any factors for stratification. To reduce predictability of a random sequence, details of any planned restriction (eg, blocking) should be provided in a separate document that is unavailable to those who enrol participants or assign interventions	-
Allocation concealment mechanism	16b	Mechanism of implementing the allocation sequence (eg, central telephone; sequentially numbered, opaque, sealed envelopes), describing any steps to conceal the sequence until interventions are assigned	-
Implementation	16c	Who will generate the allocation sequence, who will enrol participants, and who will assign participants to interventions	-
Blinding (masking)	17a	Who will be blinded after assignment to interventions (eg, trial participants, care providers, outcome assessors, data analysts), and how	-
	17b	If blinded, circumstances under which unblinding is permissible, and procedure for revealing a participant's allocated intervention during the trial	-
**Methods: Data collection, management, and analysis**			
Data collection methods	18a	Plans for assessment and collection of outcome, baseline, and other trial data, including any related processes to promote data quality (eg, duplicate measurements, training of assessors) and a description of study instruments (eg, questionnaires, laboratory tests) along with their reliability and validity, if known. Reference to where data collection forms can be found, if not in the protocol	9
	18b	Plans to promote participant retention and complete follow-up, including list of any outcome data to be collected for participants who discontinue or deviate from intervention protocols	-

Data management	19	Plans for data entry, coding, security, and storage, including any related processes to promote data quality (eg, double data entry; range checks for data values). Reference to where details of data management procedures can be found, if not in the protocol	9
Statistical methods	20a	Statistical methods for analysing primary and secondary outcomes. Reference to where other details of the statistical analysis plan can be found, if not in the protocol	13
	20b	Methods for any additional analyses (eg, subgroup and adjusted analyses)	-
	20c	Definition of analysis population relating to protocol non-adherence (eg, as randomised analysis), and any statistical methods to handle missing data (eg, multiple imputation)	-
**Methods: Monitoring**			
Data monitoring	21a	Composition of data monitoring committee (DMC); summary of its role and reporting structure; statement of whether it is independent from the sponsor and competing interests; and reference to where further details about its charter can be found, if not in the protocol. Alternatively, an explanation of why a DMC is not needed	-
	21b	Description of any interim analyses and stopping guidelines, including who will have access to these interim results and make the final decision to terminate the trial	-
Harms	22	Plans for collecting, assessing, reporting, and managing solicited and spontaneously reported adverse events and other unintended effects of trial interventions or trial conduct	-
Auditing	23	Frequency and procedures for auditing trial conduct, if any, and whether the process will be independent from investigators and the sponsor	-
**Ethics and dissemination**			
Research ethics approval	24	Plans for seeking research ethics committee/institutional review board (REC/IRB) approval	2-3, 18
Protocol amendments	25	Plans for communicating important protocol modifications (eg, changes to eligibility criteria, outcomes, analyses) to relevant parties (eg, investigators, REC/IRBs, trial participants, trial registries, journals, regulators)	-

For peer review only - http://bmjopen.bmj.com/site/about/guidelines.xhtml

Consent or assent	26a	Who will obtain informed consent or assent from potential trial participants or authorised surrogates, and how (see Item 32)	9
	26b	Additional consent provisions for collection and use of participant data and biological specimens in ancillary studies, if applicable	-
Confidentiality	27	How personal information about potential and enrolled participants will be collected, shared, and maintained in order to protect confidentiality before, during, and after the trial	14
Declaration of interests	28	Financial and other competing interests for principal investigators for the overall trial and each study site	
Access to data	29	Statement of who will have access to the final trial dataset, and disclosure of contractual agreements that limit such access for investigators	18
Ancillary and post-trial care	30	Provisions, if any, for ancillary and post-trial care, and for compensation to those who suffer harm from trial participation	-
Dissemination policy	31a	Plans for investigators and sponsor to communicate trial results to participants, healthcare professionals, the public, and other relevant groups (eg, via publication, reporting in results databases, or other data sharing arrangements), including any publication restrictions	18
	31b	Authorship eligibility guidelines and any intended use of professional writers	-
	31c	Plans, if any, for granting public access to the full protocol, participant-level dataset, and statistical code	-
**Appendices**			
Informed consent materials	32	Model consent form and other related documentation given to participants and authorised surrogates	Additional file 4
Biological specimens	33	Plans for collection, laboratory evaluation, and storage of biological specimens for genetic or molecular analysis in the current trial and for future use in ancillary studies, if applicable	-

*It is strongly recommended that this checklist be read in conjunction with the SPIRIT 2013 Explanation & Elaboration for important clarification on the items. Amendments to the protocol should be tracked and dated. The SPIRIT checklist is copyrighted by the SPIRIT Group under the Creative Commons "Attribution-NonCommercial-NoDerivs 3.0 Unported" license.

BMJ Open

Prevalence of reproductive tract infections including sexually transmitted infections among married women in urban and peri urban mid to low socioeconomic neighbourhoods of Delhi, North India, an observational study protocol

Journal:	*BMJ Open*
Manuscript ID	bmjopen-2021-059583.R1
Article Type:	Protocol
Date Submitted by the Author:	11-Feb-2022
Complete List of Authors:	Dhabhai, Neeta; Centre for Health Research and Development Society for Applied Studies<br>Chaudhary, Ritu; Centre for Health Research and Development Society for Applied Studies, Society for Applied Studies<br>Wi, Teodora; World Health Organization<br>Mburu , Gitau ; World Health Organization, 5. Department of Sexual and Reproductive Health and Research (SHR)<br>Chowdhury, Ranadip; Centre for Health Research and Development Society for Applied Studies,<br>More, Deepak; Centre for Health Research and Development Society for Applied Studies,<br>Chatterjee, Leena; Dr Dangs Lab LLP<br>De, Devjani; Dr Dangs Lab LLP<br>Kabra, Rita ; World Health Organization, Reproductive Health and Research<br>Kiarie, James; World Health Organization<br>Habib, Ndema; World Health Organization, Department of Sexual and Reproductive Health and Research including UNDP/UNFPA/UNICEF/WHO/World Bank Special Programme of Research, Development and Research Training in Human Reproduction (HRP)<br>Dang, Arjun; Dr Dangs Lab LLP<br>Dang, Manvi; Dr Dangs Lab LLP<br>Mazumder, Sarmila; Centre for Health Research and Development Society for Applied Studies
**Primary Subject Heading**:	Reproductive medicine
Secondary Subject Heading:	Reproductive medicine, Sexual health
Keywords:	REPRODUCTIVE MEDICINE, SEXUAL MEDICINE, Community gynaecology < GYNAECOLOGY

SCHOLARONE™
Manuscripts

Prevalence of reproductive tract infections including sexually transmitted infections among married women in urban and peri urban mid to low socioeconomic neighbourhoods of Delhi, North India, an observational study protocol

Neeta Dhabhai[1], Ritu Chaudhary[1], Teodora Elvira WI[2], Gitau Mburu [2], Ranadip Chowdhury[1], Deepak More[1], Leena Chatterjee[3], Devjani De[3], Rita Kabra[2], James Kiarie[2], Ndema Abu Habib[2], Arjun Dang[3], Manvi Dang[3], Sarmila Mazumder[1]

[1]Centre for Health Research and Development, Society for Applied Studies, New Delhi, India.

[2] UNDP-UNFPA-UNICEF-WHO-World Bank Special Programme of Research, Development and Research Training in Human Reproduction (HRP), Department of Sexual and Reproductive Health and Research, World Health Organization, 20 Avenue Appia, 1211 Geneva, Switzerland

[3]Dr. Dangs Lab, New Delhi, India

Corresponding author

Sarmila Mazumder, Senior Scientist, Centre for Health Research and Development, Society for Applied Studies, 45 Kalu Sarai, New Delhi-110016, India. Email: sarmila.mazumder@sas.org.in

Tel: +91 11 46043751-55; Fax: +91 11 46043756

Word count: Abstract: 300; Manuscript: 3342; References: 26; Tables: 1

For peer review only

ABSTRACT

Introduction

The Global Health Sector Strategy (GHSS) on sexually transmitted infections (STI), endorsed by the World Health Assembly in 2016 aims to end STIs as public health threat by 2030. WHO conducts global estimates of prevalence to monitor progress towards achieving the same. However, limited laboratory confirmed data exists of STIs and reproductive tract infections (RTI) apart from few prevalence surveys among key populations and clinic-based reports, including in India. Syndromic approach is the cornerstone of RTI/STI management and to maximize the diagnostic accuracy, there is a need to determine the main aetiologies of vaginal discharge. This study aims to estimate the prevalence of common STIs and RTIs and their aetiological organisms in symptomatic and asymptomatic women living in the urban and peri-urban, mid- to low-socioeconomic neighbourhoods of Delhi, North India.

Methods and analysis

A cross-sectional study will be conducted among 440 married women who participated in the 'Women and Infants Integrated Interventions for Growth Study (WINGS). Information on socio-demographic profile, sexual and reproductive health will be collected, followed by examination and collection of vaginal swabs for Nucleic acid amplification tests (NAAT) to diagnose Neisseria gonorrhoeae, Chlamydia trachomatis, and Trichomonas vaginalis and microscopy to identify Bacterial Vaginosis and Candida albicans. Treatment will be as per the syndromic approach recommendations in the Indian National Guidelines. Data will be analysed to estimate prevalence, presence of symptoms, and signs associated with laboratory confirmed RTIs/STIs using STATA version 16.0 (StataCorp).

Ethics and dissemination

This study protocol has been approved by the ethics review committees of the World Health Organization and Society for Applied Studies (SAS/ERC/RHR-RTI/STI/2020). Approval has been obtained by the WINGS investigators from Society for Applied Studies (SAS) ERC to

For peer review only

share the contact details of the participants with the investigators. The findings will be published in peer-reviewed journals and disseminated through scientific conferences.

Trial Registration: CTRI/2020/03/023954

For peer review only

Strengths and limitations of this study

- Recent data on the prevalence of RTI/STI and aetiologies of symptomatic RTI/STI in India are limited; this study responds to this research gap.
- It will provide crucial information on prevalence of laboratory confirmed NG, CT and TV among asymptomatic woman, in addition to those with symptoms.
- This study addresses the WHO's recommendation to periodically ascertain the etiology of vaginal discharge syndrome every 2 to 3 years in-order to update or modify the national guideline.
- This is not a community-based prevalence survey, which would require randomized sampling and would be costly.
- Participants in this study are limited to a prespecified cohort of women hence, generalisability of the findings and conclusions will need to be interpreted with care.

For peer review only

INTRODUCTION

Sexually transmitted infections (STI) and reproductive tract infections (RTI) are a significant cause of global burden of disease.[1] Of the eight pathogens of highest public health importance, four are curable, Treponema pallidum (TP), Neisseria gonorrhoeae (NG), Chlamydia trachomatis (CT), and Trichomonas vaginalis (TV) while the other four of viral aetiology are not, Hepatitis B, Herpes simplex virus (HSV), Human Immunodeficiency Virus (HIV), and Human papillomavirus (HPV).[2 3] Globally, 374 million new infections of the four curable STIs occur annually.[2 3] These infections have significant public health consequences including infertility,[4 5] ectopic pregnancies[6 7] prematurity and neonatal deaths,[8] as well as increased risk of HIV acquisition.[9 10] Among RTIs, Bacterial vaginosis (BV) is of emerging importance as it reflects the dysbiotic state of the vaginal microbiome and increases susceptibility to other STIs.[3 4] Candidial genital infection is the leading cause of fungal vulvo-vaginitis and asymptomatic microorganism colonization can occur in up to 60% of the cases.[8]

Given the importance of STIs and RTIs, strengthening epidemiological data to inform management and control is imperative. In its global health strategy for the control of STIs and tool for strengthening surveillance of STIs, the World Health Organization (WHO) emphasizes on four key elements, including case reporting, prevalence assessment, aetiologies, and monitoring of antimicrobial resistance[11]. Global control and prevention of STIs will contribute to progress towards achievement of the Sustainable Development Goals to ensure universal access to sexual and reproductive health-care services.[2]

To address the limited national data, WHO developed the STI spectrum model to assist countries to estimate the prevalence. Recent estimates suggest that incidence of STIs has plateaued at 1 million cases annually;[9] however, significant regional heterogeneity regarding the burden and availability of data exists especially in Asia and Oceania.[9] In India, there is no recent data to determine the magnitude of STIs. Few prevalence surveys among key populations and pregnant women exist, to inform national STI estimates. Regular case reporting of infections

For peer review only

such as gonorrhoea is available, but are under-reported, thus the generation of more accurate prevalence data among the general population is needed. Apart from regular case reporting, WHO recommends studies to determine local aetiologies of STIs every 2 to 3 years to improve management.[10]

In India, it is estimated that 6% of the adult population is infected with one or more STIs at any one time. Due to limited access and affordability of STI laboratory diagnosis at the primary level of care where majority of STI cases seek care, syndromic case management remains the cornerstone of STI control.[12] This, as is the case in many low- and middle-income countries is partly due to its lower infrastructural/equipment investments and limited availability of expertise. Syndromic approach is based on presence of signs and symptoms reported, and provides immediate treatment for the most common organisms causing the identified syndrome through the use of pre-packaged colour coded drug kits. It is also recommended for the pregnant women.

Based on new evidence, WHO recommends quality-assured molecular assays for treatment of people with symptoms of NG, CT and TV. If quality-assured molecular assays are not available, treatment based syndromic approach is recommended.[13] However, syndromic approach has certain limitations. It results in over treatment or missed diagnosis of cervical infections (NG and/or CT) compared with aetiological approach using molecular assay.[14 15] While aetiological determination of STI is more accurate, it is often not feasible in most health facilities. Aetiological data among symptomatic and asymptomatic women is inadequate across India.[14] Of the few studies that have explored aetiologies of vaginal discharge, the focus has been among sex workers in 2003 and 2011,[14 15] and these studies found high rates of STIs, especially NG and CT. In 2009, a comparative study of syndromic and etiologic diagnosis of STIs was conducted among general population in Delhi[16] that included symptomatic and asymptomatic pregnant and non-pregnant women. It revealed a high proportion of women complaining of vaginal discharge, self-reporting of morbidity (65%) and presence of STD-related syndrome

For peer review only

(71.4%). The aetiological microorganism was established in 32.2% of the cases. Our study intends to build upon these findings to generate more detailed quality-assured data related to aetiologies of RTI/STIs among symptomatic and asymptomatic women.

For peer review only

METHODS

Aims and objectives

The overall aim of the study is to generate information and data related to RTI/STIs among women in India. More specifically, there are three objectives. First, to determine the prevalence of NG, CT, TV, BV and Candida albicans (CA) among women residing in the peri-urban and urban mid to low socioeconomic areas of South Delhi at least 14 days after they exit from WINGS. Second, to assess the diagnostic validity of the current RTI/STI syndromic case management practice relative to the etiologic diagnosis based on laboratory confirmation of NG*, CT, TV, BV and CA* among symptomatic and asymptomatic women. Third, to identify demographic, behavioral, sexual and clinical factors associated with laboratory confirmed RTI/STI among women. Similar data on Syphilis is already being collected as a part of WINGS.[17]

Study design and participants

This is a cross sectional independent study enrolling women at least 14 days after their exit from WINGS. WINGS was an individually-randomized factorial trial to assess impact of a package of community level health, nutrition, WASH and psychosocial care interventions delivered in preconception, during pregnancy and in the first 2 years after birth, on birth outcomes and infant growth. 13500, married women aged 18-30 years, living with their husband with no or one child, and wanting more children, were enrolled and randomized to receive a pre-conception intervention package or routine care, until pregnant or 18 months post enrolment. At pregnancy confirmation, second randomization was done to receive pregnancy and early childhood interventions or routine care. This resulted in four groups of women that received (1) preconception, pregnancy and early childhood interventions, (2) only pre-conception interventions, (3) only pregnancy and early childhood intervention, and (4) no interventions

Women will be exiting WINGS at two time points: at 18 months since enrolment for women who do not get pregnant, or at 24 months post-delivery for women who get pregnant. A more detailed protocol for WINGS was published elsewhere.[17]

Sample size

Overall, 6% of adults in India are estimated to have STIs.[14] Existing studies suggest that 32% of cases have laboratory confirmed STI.[16]Therefore, a sample size of 440 women will be adequate to ascertain the prevalence of aetiological diagnosis of STI in symptomatic and asymptomatic women with 95% confidence, 15% relative precision and 20% non-response rate allowance.

Study procedures

Study preparatory activity started in Jan 2021 and is expected to be completed in December 2022

Recruitment

Study participants who exit from WINGS will be contacted after at least 14 days to ascertain their willingness to participate and those who agree will be offered to take part in the study.

Informed consent

Written individual informed consent will be obtained from all study participants at enrolment for quantitative data collection, collection of endocervical swabs and for interviews by research assistants at the study clinic. For those who are unable to read, the consent form will be read out by the worker. Those participants who are unable to sign, a thumb imprint will be taken and witnessed (counter signed) by an impartial literate witness. The informed consent form will be translated into simple Hindi language that can be easily read and understood.

Inclusion Criteria

Women who exit WINGS study, give consent and are eligible for sampling Eligibility is ascertained through screening. Women are screened out and visit rescheduled if they are menstruating at the time of screening, have history of vaginal douching, usage of any vaginal

For peer review only

tablets or cream and had sexual intercourse in the last 48 hours. Blood, semen or medication can potentially interfere with test results.

Exclusion Criteria

Women who exit WING study but decline to consent

Data collection

Using a structured questionnaire, the research assistants will conduct face-to-face interviews to collect information on socio-demographic, economic and sexual behaviour. The sociodemographic and economic questionnaire includes information on educational status, occupation, family size and structure, annual income, number of family members, religion, household assets, health insurance cover and WASH practices. Questionnaire on sexual behaviour includes information on age of first intercourse, number of partners, use of barrier methods, symptoms of RTI/STI in previous one week, history of RTI/STIs and treatment received during last three months, among other data.

Gynaecological examination

A thorough history and genital and pelvic examination including abdominal palpation will be done by the study gynaecologist with aseptic precautions in complete privacy, for all participants irrespective of the symptoms. With the participant in the dorsal lithotomy position, a visual inspection of the external genitalia, followed by a per speculum and bimanual examination will be done.

Signs of RTI/ STI and other observations will be documented in an electronic tracker. These will include nature of vaginal discharge (i.e.,colour, odour, consistency, amount), presence and type of cervical discharge, condition of cervix, any genital ulcers, adnexal or pelvic tenderness, size of uterus, any adnexal mass or lump. High vaginal swab and endo-cervical swab will be collected as per the standard operating procedures. Women will be provided free medication according to the diagnosis, as per the National Guidelines on RTI/STI.[18]

For peer review only

Sample collection, storage and transportation

Two swabs will be collected, one vaginal and other endocervical, by the study gynaecologist. The vaginal walls and cervix will be visualized using self-retaining sterile Cusco's speculum. Vaginal swab specimen will be collected from the posterior fornix and the lateral walls of the vagina for detection of BV and CA and kept in a pre-labelled dry collection tube. Following this, the endocervical swab from the test kit will be inserted gently into the endocervical canal, rotated and withdrawn and kept in the pre-labelled transport reagent container for Nucleic Acid Amplification test (NAAT) for CT, NG and TV.

All samples will be stored at 2-8°C in the laboratory and transportation to the testing laboratory will be done daily, in cool boxes maintained at 2-8°C.

Laboratory procedures

NAAT, a real time polymerase chain reaction (PCR) having the highest specificity and sensitivity (98-100%) among the available conventional tests, will be used to detect CT, NG and TV.[19] We will use Cartridge Based (CB) NAAT Gene Xpert platform which is an automated in vitro diagnostic test for qualitative detection and differentiation of DNA of CT, NG and TV and is performed on Cepheid GeneXpert Instrument Systems.[20] With this we will use the GeneXpert CT/NG Cepheid In vitro diagnostic (IVD), and GeneXpert TV Cepheid IVD kits.[21]

The GeneXpert Instrument Systems automate and integrate DNA extraction, amplification, and detection of the target sequences in simple or complex samples using Reverse transcription (RT-PCR). The systems consist of an instrument connected with a computer, with preloaded software for running tests and viewing and generating results. The systems require single-use disposable cartridges that hold the PCR reagents and host the PCR process.

Reagents for the detection of a Sample Processing Control (SPC), a Sample Adequacy Control (SAC), and a Probe Check Control (PCC) are also included in the cartridge. The SPC is present to control for adequate processing of the target bacteria and to monitor the presence of inhibitors in the PCR reaction.

For peer review only

The SAC reagents detect the presence of a single copy human gene and monitor whether the sample contains human DNA. The PCC verifies reagent rehydration, PCR tube filling in the cartridge probe integrity, and dye stability. The primers and probes in the Xpert CT/NG test detect chromosomal sequences in the bacteria. One target is detected for CT (CT1) and two different targets are detected for NG (NG2 and NG4). Both NG targets need to be positive for the Xpert CT/NG test to return a positive NG result. Prepared sample is transferred to the sample chamber of the Gene expert cartridge and loaded on the GeneXpert machine and the test is performed.

Summary and detailed test results are generated by the software in approximately 90 minutes and are displayed in tabular and graphic formats. Nugent's Scoring criteria using a gram stained slide is the current 'gold standard with ~77% specificity and 90% sensitivity will be used to diagnose BV.[22]

A smear will be prepared, and gram staining done. Microscopic examination will be done to detect clue cells and semi-quantitative evaluation for Gram-positive, Gram-negative or Gram-variable bacterial morphotypes quantified. Women will be categorized as non-BV (Nugent score 0–3), intermediate (Nugent score 4–6) or BV (Nugent score ≥ 7) (Table 1).

Table 1. Nugent Score

Organism morphotype	Number/oil immersion field	Score
Lactobacillus-like (parallel sided, gram positive rods)	>30	0
	5-30	1
	1-4	2
	<1	3
	0	4
Mobiluncus-like (curved, gram negative rods)	>5	2
	<1.4	1
	0	0
Gardnerella/Bacteroides-like (tiny, gram variable coccobacilli and pleomorphic rods with vacuoles	>30	4
	5-30	3

For peer review only

Organism morphotype	Number/oil immersion field	Score
	1-4	2
	<1	1
	0	0

Total score: 0-3=Normal; 4-6=Intermediate, repeat test later; 7-10= Bacterial vaginosis

Microscopical examination of the vaginal smear will be done for yeast cells with pseudo hyphae to detect CA.

Complete reports will be generated and sent after 48 hrs.

Quality Control

For Gram stain, on each slide, a control area for Staph aureus (ATCC25923) & E. coli (ATCC25922) is put as a staining control. For CBNAAT, each test kit includes an inbuilt Sample Processing Control (SPC), a Sample Adequacy Control (SAC) and a Probe Check Control (PCC). External controls (one positive and one negative) will be used in accordance with local, state accrediting organizations, as applicable.

Kit Verification

As the Cepheid kits to be used for the study for CT/NG are FDA approved while that for TV is Research Use Only (RUO), a kit verification procedure is planned to evaluate the performance of the GeneXpert platform. A verification panel comprising of 17 samples containing known CT, NG, and TV of high, medium, and low concentration will be tested on GeneXpert using the recommended kits. The results will be compiled and reviewed to determine the suitability of the platform and the kits for diagnosis of STI in the study.

Management of RTI/STIs

Women with signs and symptoms of RTI/STIs will be treated based on the syndromic approach as recommended in the national guidelines.[11]Their partners will be notified and provided necessary treatment. The laboratory reports will be reviewed by the study gynaecologists and delivered to their homes with advice to revisit if needed. Asymptomatic women who need further

For peer review only

treatment will be invited to the study clinic for an appointment with the study gynaecologists and to receive medicines. Women will also be provided information and counselled on prevention of STI/RTI. Women requiring admission or additional management will be referred to the collaborating hospital. A complete course of treatment and supply of condoms will be provided for free.

Statistical analysis and outcome measures

Descriptive and inferential statistical analysis will be performed. Simple proportions and 95% confidence bounds will be calculated to determine prevalence for overall laboratory confirmed RTI/STI and individual micro-organism by binomial exact method. We will use a method of purposeful selection of covariates to identify variables for the multivariable models.[23 24] In the multivariable models, we will include the variables that will change the odds ratio of the outcome variables by 20% from the univariable models. We will present the adjusted models, including variables that will be identified in the procedure. We will assess whether any of the demographic, behavioural, sexual and clinical risk factors are modifying the association between laboratory conformed RTI/STI and any of the above-mentioned variables on a multiplicative scale. Computation of sensitivity, specificity, predictive values and likelihood ratios will be derived in diagnosing vaginal infection (TV, BV) and cervical infection (NG, CT) using NAAT as the gold standard of diagnosis compared with syndromic approach which is informed by the presence of symptoms and signs. Analysis will be done using STATA, version 16.0 (StataCorp).

Patient and public involvement

Participants and the public were not involved in the design of this study. However, study results will be shared with the participants and informed about their aetiological diagnosis and provided appropriate treatment.

Confidentiality

All data will be de-identified, analyzed and aggregated before reporting. Furthermore, data will

For peer review only

be stored securely and only reviewed by the immediate research team. The information collected from the study participants will be kept confidential and stored in a locked area which can only be accessed by the study team. The participants will not be identified by his/her name but only by a study number. All study documents will be stored for a period of 5 years after which they will be destroyed. Knowledge from this study will be disseminated through reports given to the ethics review committee, government agencies and publications but none will have the name of the study participants.

Tokens/compensation

Participants will be provided with a reasonable token (not exceeding USD 2) of appreciation, along with free medical examination, treatment for STI/RTI and condoms. Usually this is given to compensate for the time spent on study participation. It is considered reasonable to compensate participants for their time. The amount is not considered large to constitute an undue inducement.

Gender and social equity considerations

The proposed study addresses the sexual and reproductive rights of women related to RTI/STI and will identify risks and vulnerabilities in relation to RTI/ STIs in the study context. By understanding the prevalence of laboratory confirmed RTI/STIs as well as diagnostic validity of syndromic approach, the study will provide useful information to the national program. It will also assist women to access treatment, including asymptomatic women, which will be useful in addressing inequality to service access. The research questions are focused on women. However, there will be opportunities for sexual partners of women identified with RTI/STI to be notified and subsequently provided with treatment as per syndromic management following laboratory/ etiologic results.

Community engagement

This study will enrol women who previously participated in WINGS, which has a large component of community participation. Participants will also be contacted at their homes, and

For peer review only

local communities will continually be reached and mobilized with the results to enhance uptake of interventions that promote better woman and infant outcomes.

Research capacity strengthening

It is envisioned that exchange of information related to conduct of research and data analysis will continually take place between WHO and the research partners.

Dissemination

The result of the study will be presented in scientific conferences and published in appropriate peer-reviewed journals.

For peer review only

DISCUSSION

This study aims to fill an existing gap related to a lack of epidemiologic information on STIs in India. A recent systematic review of syndromic approach found that there was very limited validation of syndromic approach as well as etiologic studies globally.[25] With an ever-changing epidemiology of STIs, there is a need to encourage countries to regularly conduct etiologic studies of syndromes, to inform national STI case management guidelines.

This study will evaluate the diagnostic validity of RTI/STI syndromic approach among women of reproductive age in India while providing important information related to the prevalence of RTI/STIs and the aetiologies of vaginal discharge syndrome in the study context, where there is insufficient prevalence and etiologic data. Given the serious consequences of RTI/STIs on women's health, sexual and social relationships and psychological well-being[26] this research will make an important contribution to the understanding and mitigation of RTI/STIs in India. Data on prevalence will be relevant for improving the national STI case management in India, and for planning RTI/STI services for women, in order to reduce maternal and neonatal morbidity and mortality, adverse birth outcomes, and the sequelae of these infections. However the generalisability of the findings from this study to wider population may be limited by the fact that women who participate in research studies self-select, and therefore may bias the prevalence results

In context of high volume of specimens, one of the key challenges for this study will be to ensure quality of sample collection, transport and processing which will be mitigated through training and regular monitoring. While important, our primary intention at this point is not to compare RTI/STI prevalence between two groups of women (those who get pregnant and those who do not). This sub-study will not collect data related to syphilis since prevalence data for on Syphilis is already being collected as a part of WINGS.[16]

For peer review only

Abbreviations

GHSS; Global Health Sector Strategy, **RTI;** Reproductive tract infections, **STI;** Sexually transmitted infections, **WINGS;** Women and Infants Integrated Interventions for Growth Study, **NABL;** National Accreditation Board for Testing and Calibration, **NAAT;** Nucleic acid amplification tests, **NG;** Neisseria gonorrhoeae, **CT**; Chlamydia trachomatis, **TV**; Trichomonas vaginalis, **BV;** Bacterial Vaginosis, **CA;** Candida albicans, **TP;** Treponema pallidum, **HSV**; Herpes simplex virus, **HIV;** Human Immunodeficiency Virus, **HPV;** Human papillomavirus, **WHO;** World Health Organization, **LMP;** last menstrual period, **PCR;** Polymerase chain reaction, **SPC;** Sample Processing Control, **SAC;** Sample Adequacy Control, **PCC;** Probe Check Control, **RUO;** Research use only, **ERC:** Ethics Research Committee, **WASH**: Water, Sanitation and Hygiene.

Acknowledgements

This study is funded by WHO Department of Sexual and Reproductive Health and Research. WHO/SRH department includes the UNDP-UNFPA-UNICEF-WHO-World Bank Special Programme of Research, Development and Research Training in Human Reproduction (HRP). Authors thank Dr Dang's laboratory, implementation partners of the WINGS study (Bills and Melinda Gates Foundation (BMGF), the department of Biotechnology and the Government of India through Biotechnology Industry Research Assistance Council (BIRAC) including CHRD-SAS, Safdarjung Hospital and the WHO Department of Maternal, Newborn, Child and Adolescent health (MCA). Views expressed are those of the authors and not necessarily those of their respective institutions.

Authors' contributions

JK, TAW, GM, RK, SM, ND, conceived the study. TAW, GM, SM, ND, RC, NAH and DM prepared the protocol. All authors reviewed and approved the final manuscript.

Funding

For peer review only

This study is funded by WHO Department of Sexual and Reproductive Health and Research. WHO/SRH Department includes the UNDP-UNFPA-UNICEF-WHO-World Bank Special Programme of Research, Development and Research Training in Human Reproduction (HRP). Grant number - 202553792. The funding agency has no role in the design of the current protocol and will not have any role in the implementation, analysis and reporting of the study results.

Competing interests

None declared

Patient consent for publication

Not required

Ethics and dissemination

Approval for this study has been obtained from the Ethics Review Committee of the Centre for Health Research and Development, Society for Applied Studies (SAS/ERC/RHR-RTI/STI/2020) and the World Health Organization, Geneva (A-ID: A65999). Approval has been obtained by the WINGS investigators from SAS ERC to share the contact details of the participants with the investigators. The findings will be published in peer-reviewed journals and disseminated through scientific conferences.

Data availability statement

The data underlying this article will be shared on reasonable request to the corresponding author. Data arising from the study will be kept by SAS for 5 years and anonymized forms of these data will be available on request from the corresponding author.

Declaration of interests

The authors declare no competing interests.

REFERENCES

1. WHO. Report on global sexually transmitted infection surveillance, 2018. Geneva: World Health Organization. 2018

2. World Health Organization. Global progress report on HIV, viral hepatitis and sexually transmitted infections.*Accountability for the global health sector strategies 2016–2021* Geneva: World Health Organization

3. Newman L, Rowley J, Vander Hoorn S, et al. Global Estimates of the Prevalence and Incidence of Four Curable Sexually Transmitted Infections in 2012 Based on Systematic Review and Global Reporting. *PLOS ONE* 2015;10(12):e0143304. doi: 10.1371/journal.pone.0143304

4. Bautista CT, Wurapa E, Sateren WB, et al. Bacterial vaginosis: a synthesis of the literature on etiology, prevalence, risk factors, and relationship with chlamydia and gonorrhea infections. *Mil Med Res* 2016;3:4-4. doi: 10.1186/s40779-016-0074-5

5. Bertini M, Serdaroglu S, Kutlubay Z. Bacterial vaginosis and sexually transmitted diseases: relationship and management. *Fundamentals of Sexually Transmitted Infections* 2017;75

6. Tankhiwale, S.S., Sharma, M.K., Surpam, R.B., & Fussey, S. Etiological study of vaginal discharge syndrome in RTI clinic attendees in a tertiary care hospital. *The New Indian Journal of OBGYN*. 2017; 4(1):50-3. DOI:10.21276/OBGYN.2017.4.1.11

7. Siracusano S, Silvestri T, Casotto D. Sexually transmitted diseases: epidemiological and clinical aspects in adults. *Urologia* 2014;81(4):200-8. doi: 10.5301/uro.5000101 [published Online First: 2014/12/24]

8. Sustr V, Foessleitner P, Kiss H, et al. Vulvovaginal Candidosis: Current Concepts, Challenges and Perspectives. *Journal of fungi (Basel, Switzerland)* 2020;6(4) doi: 10.3390/jof6040267 [published Online First: 2020/11/12]

For peer review only

9. Rowley J, Vander Hoorn S, Korenromp E, et al. Chlamydia, gonorrhoea, trichomoniasis and syphilis: global prevalence and incidence estimates, 2016. *Bulletin of the World Health Organization* 2019;97(8):548-62p. doi: 10.2471/blt.18.228486 [published Online First: 2019/08/07]

10. Department of Reproductive Health and Research, World Health Organization, Strategies and laboratory methods for strengthening surveillance of sexually transmitted infection 2012, https://www.who.int/reproductivehealth/publications/rtis/9789241504478/en/

11. World Health Organization. A tool for strengthening STI surveillance at the country level. 2015, https://www.who.int/reproductivehealth/publications/rtis/sti-surveillance/en/

12. National guidelines on prevention, management and control of reproductive tract infections including sexually transmitted infections.2006 http://naco.gov.in/sites/default/files/National_Guidelines_on_PMC_of_RTI_Including_STI%201.pdf

13. World Health Organization, Guidelines for the management of symptomatic sexually transmitted infections. Geneva: World Health Organization; 2021. Licence: CC BY-NC-SA 2021, https://www.who.int/publications/i/item/9789240024168

14. Desai VK, Kosambiya JK, Thakor HG, et al. Prevalence of sexually transmitted infections and performance of STI syndromes against aetiological diagnosis, in female sex workers of red light area in Surat, India. *Sexually transmitted infections* 2003;79(2):111-5. doi: 10.1136/sti.79.2.111 [published Online First: 2003/04/12]

15. Das A, Prabhakar P, Narayanan P, et al. Prevalence and assessment of clinical management of sexually transmitted infections among female sex workers in two cities of India. *Infectious diseases in obstetrics and gynecology* 2011;2011:494769. doi: 10.1155/2011/494769 [published Online First: 2011/07/13]

16. Ray K, Muralidhar S, Bala M, et al. Comparative study of syndromic and etiological diagnosis of reproductive tract infections/sexually transmitted infections in women in Delhi.

For peer review only

International journal of infectious diseases : IJID : official publication of the International Society for Infectious Diseases 2009;13(6):e352-9. doi: 10.1016/j.ijid.2008.11.021 [published Online First: 2009/02/25]

17. Taneja S, Chowdhury R, Dhabhai N, et al. Impact of an integrated nutrition, health, water sanitation and hygiene, psychosocial care and support intervention package delivered during the pre- and peri-conception period and/or during pregnancy and early childhood on linear growth of infants in the first two years of life, birth outcomes and nutritional status of mothers: study protocol of a factorial, individually randomized controlled trial in India. *Trials* 2020;21(1):127. doi: 10.1186/s13063-020-4059-z [published Online First: 2020/02/02]
18. Department of AIDS Control, Ministry of Health and Family Welfare. Governmentof India. National Guidelines on Prevention, Management and Control of Reproductive Tract Infections and Sexually Transmitted Infections. 2014. Available at: http://naco.gov.in/sites/default/files/National%20RTI%20STI%20technical%20guidelines%20Sep2014_0.pdf
19. Susanne Jacobsson, Iryna Boiko, Daniel Golparian, Karel Blondeel, James Kiarie, Igor Toskin, Rosanna W. Peeling, Magnus Unemo WHO laboratory validation of Xpert® CT/NG and Xpert® TV on the GeneXpert system verifies high performances *APMIS* 2018; 126: 907– 912. doi: https://doi.org/10.1111/apm.12902
20. CBNAAT Gene Xpert platform is an automated in vitro C and differentiation of DNA from CT, NG. https://www.cepheid.com/en/systems/GeneXpert-Family-of-Systems/GeneXpert-System
21. The GeneXpert CT/NG Cepheid IVD, and GeneXpert used to detect CT, NG, and TV. https://www.cepheid.com/en/tests/Sexual-Health/Xpert-CT-NG
22. Cox C, McKenna JP, Watt AP, et al. New assay for Gardnerella vaginalis loads correlates with Nugent scores and has potential in the diagnosis of bacterial vaginosis. Journal of

For peer review only

medical microbiology 2015;64(9):978-84. doi: 10.1099/jmm.0.000118 [published Online First: 2015/08/25]

23. Bursac Z, Gauss CH, Williams DK, et al. Purposeful selection of variables in logistic regression. *Source code for biology and medicine* 2008;3:17. doi: 10.1186/1751-0473-3-17 [published Online First: 2008/12/18]

24. Hosmer Jr DW, Lemeshow S, Sturdivant RX. Applied logistic regression: John Wiley & Sons 2013.

25. Zemouri C, Wi TE, Kiarie J, et al. The Performance of the Vaginal Discharge Syndromic Management in Treating Vaginal and Cervical Infection: A Systematic Review and Meta-Analysis. *PLoS One* 2016;11(10):e0163365. doi: 10.1371/journal.pone.0163365 [published Online First: 2016/10/06]

26. Cooperman NA, Shastri JS, Shastri A, et al. HIV prevalence, risk behavior, knowledge, and beliefs among women seeking care at a sexually transmitted infection clinic in Mumbai, India. *Health care for women international* 2014;35(10):1133-47. doi: 10.1080/07399332.2013.770004 [published Online First: 2013/05/11]

For peer review only

STANDARD PROTOCOL ITEMS: RECOMMENDATIONS FOR INTERVENTIONAL TRIALS

SPIRIT 2013 Checklist: Recommended items to address in a clinical trial protocol and related documents*

Section/item	Item No	Description	Addressed on page number
**Administrative information**			
Title	1	Descriptive title identifying the study design, population, interventions, and, if applicable, trial acronym	1
Trial registration	2a	Trial identifier and registry name. If not yet registered, name of intended registry	3
	2b	All items from the World Health Organization Trial Registration Data Set	-
Protocol version	3	Date and version identifier	-
Funding	4	Sources and types of financial, material, and other support	19
Roles and responsibilities	5a	Names, affiliations, and roles of protocol contributors	18
	5b	Name and contact information for the trial sponsor	19
	5c	Role of study sponsor and funders, if any, in study design; collection, management, analysis, and interpretation of data; writing of the report; and the decision to submit the report for publication, including whether they will have ultimate authority over any of these activities	19
	5d	Composition, roles, and responsibilities of the coordinating centre, steering committee, endpoint adjudication committee, data management team, and other individuals or groups overseeing the trial, if applicable (see Item 21a for data monitoring committee)	-

For peer review only

**Introduction**			
Background and rationale	6a	Description of research question and justification for undertaking the trial, including summary of relevant studies (published and unpublished) examining benefits and harms for each intervention	5-7
	6b	Explanation for choice of comparators	-
Objectives	7	Specific objectives or hypotheses	8
Trial design	8	Description of trial design including type of trial (eg, parallel group, crossover, factorial, single group), allocation ratio, and framework (eg, superiority, equivalence, noninferiority, exploratory)	8
**Methods: Participants, interventions, and outcomes**			
Study setting	9	Description of study settings (eg, community clinic, academic hospital) and list of countries where data will be collected. Reference to where list of study sites can be obtained	8
Eligibility criteria	10	Inclusion and exclusion criteria for participants. If applicable, eligibility criteria for study centres and individuals who will perform the interventions (eg, surgeons, psychotherapists)	9-10
Interventions	11a	Interventions for each group with sufficient detail to allow replication, including how and when they will be administered	-
	11b	Criteria for discontinuing or modifying allocated interventions for a given trial participant (eg, drug dose change in response to harms, participant request, or improving/worsening disease)	-
	11c	Strategies to improve adherence to intervention protocols, and any procedures for monitoring adherence (eg, drug tablet return, laboratory tests)	-
	11d	Relevant concomitant care and interventions that are permitted or prohibited during the trial	13-14
Outcomes	12	Primary, secondary, and other outcomes, including the specific measurement variable (eg, systolic blood pressure), analysis metric (eg, change from baseline, final value, time to event), method of aggregation (eg, median, proportion), and time point for each outcome. Explanation of the clinical relevance of chosen efficacy and harm outcomes is strongly recommended	-
Participant timeline	13	Time schedule of enrolment, interventions (including any run-ins and washouts), assessments, and visits for participants. A schematic diagram is highly recommended (see Figure)	8

For peer review only - http://bmjopen.bmj.com/site/about/guidelines.xhtml

Sample size	14	Estimated number of participants needed to achieve study objectives and how it was determined, including clinical and statistical assumptions supporting any sample size calculations	9
Recruitment	15	Strategies for achieving adequate participant enrolment to reach target sample size	9
**Methods: Assignment of interventions (for controlled trials)**			
Allocation:			
Sequence generation	16a	Method of generating the allocation sequence (eg, computer-generated random numbers), and list of any factors for stratification. To reduce predictability of a random sequence, details of any planned restriction (eg, blocking) should be provided in a separate document that is unavailable to those who enrol participants or assign interventions	-
Allocation concealment mechanism	16b	Mechanism of implementing the allocation sequence (eg, central telephone; sequentially numbered, opaque, sealed envelopes), describing any steps to conceal the sequence until interventions are assigned	-
Implementation	16c	Who will generate the allocation sequence, who will enrol participants, and who will assign participants to interventions	-
Blinding (masking)	17a	Who will be blinded after assignment to interventions (eg, trial participants, care providers, outcome assessors, data analysts), and how	-
	17b	If blinded, circumstances under which unblinding is permissible, and procedure for revealing a participant's allocated intervention during the trial	-
**Methods: Data collection, management, and analysis**			
Data collection methods	18a	Plans for assessment and collection of outcome, baseline, and other trial data, including any related processes to promote data quality (eg, duplicate measurements, training of assessors) and a description of study instruments (eg, questionnaires, laboratory tests) along with their reliability and validity, if known. Reference to where data collection forms can be found, if not in the protocol	10
	18b	Plans to promote participant retention and complete follow-up, including list of any outcome data to be collected for participants who discontinue or deviate from intervention protocols	-

Data management	19	Plans for data entry, coding, security, and storage, including any related processes to promote data quality (eg, double data entry; range checks for data values). Reference to where details of data management procedures can be found, if not in the protocol	14-15
Statistical methods	20a	Statistical methods for analysing primary and secondary outcomes. Reference to where other details of the statistical analysis plan can be found, if not in the protocol	14
	20b	Methods for any additional analyses (eg, subgroup and adjusted analyses)	-
	20c	Definition of analysis population relating to protocol non-adherence (eg, as randomised analysis), and any statistical methods to handle missing data (eg, multiple imputation)	-
**Methods: Monitoring**			
Data monitoring	21a	Composition of data monitoring committee (DMC); summary of its role and reporting structure; statement of whether it is independent from the sponsor and competing interests; and reference to where further details about its charter can be found, if not in the protocol. Alternatively, an explanation of why a DMC is not needed	-
	21b	Description of any interim analyses and stopping guidelines, including who will have access to these interim results and make the final decision to terminate the trial	-
Harms	22	Plans for collecting, assessing, reporting, and managing solicited and spontaneously reported adverse events and other unintended effects of trial interventions or trial conduct	-
Auditing	23	Frequency and procedures for auditing trial conduct, if any, and whether the process will be independent from investigators and the sponsor	-
**Ethics and dissemination**			
Research ethics approval	24	Plans for seeking research ethics committee/institutional review board (REC/IRB) approval	2-3, 19
Protocol amendments	25	Plans for communicating important protocol modifications (eg, changes to eligibility criteria, outcomes, analyses) to relevant parties (eg, investigators, REC/IRBs, trial participants, trial registries, journals, regulators)	-

For peer review only - http://bmjopen.bmj.com/site/about/guidelines.xhtml

Consent or assent	26a	Who will obtain informed consent or assent from potential trial participants or authorised surrogates, and how (see Item 32)	9
	26b	Additional consent provisions for collection and use of participant data and biological specimens in ancillary studies, if applicable	-
Confidentiality	27	How personal information about potential and enrolled participants will be collected, shared, and maintained in order to protect confidentiality before, during, and after the trial	14-15
Declaration of interests	28	Financial and other competing interests for principal investigators for the overall trial and each study site	
Access to data	29	Statement of who will have access to the final trial dataset, and disclosure of contractual agreements that limit such access for investigators	19
Ancillary and post-trial care	30	Provisions, if any, for ancillary and post-trial care, and for compensation to those who suffer harm from trial participation	-
Dissemination policy	31a	Plans for investigators and sponsor to communicate trial results to participants, healthcare professionals, the public, and other relevant groups (eg, via publication, reporting in results databases, or other data sharing arrangements), including any publication restrictions	19
	31b	Authorship eligibility guidelines and any intended use of professional writers	-
	31c	Plans, if any, for granting public access to the full protocol, participant-level dataset, and statistical code	-
**Appendices**			
Informed consent materials	32	Model consent form and other related documentation given to participants and authorised surrogates	Consent form attached
Biological specimens	33	Plans for collection, laboratory evaluation, and storage of biological specimens for genetic or molecular analysis in the current trial and for future use in ancillary studies, if applicable	-

*It is strongly recommended that this checklist be read in conjunction with the SPIRIT 2013 Explanation & Elaboration for important clarification on the items. Amendments to the protocol should be tracked and dated. The SPIRIT checklist is copyrighted by the SPIRIT Group under the Creative Commons "Attribution-NonCommercial-NoDerivs 3.0 Unported" license.

For peer review only - http://bmjopen.bmj.com/site/about/guidelines.xhtml
